# NLRP3 deficiency aggravated DNFB-induced chronic itch by enhancing type 2 immunity IL-4/TSLP-TRPA1 axis in mice

**DOI:** 10.3389/fimmu.2024.1450887

**Published:** 2025-01-10

**Authors:** Si-Ting Huang, Zuo-Ming Chen, Zhe Peng, Yu Wang, Fan Yang, Yang Tang, Zi Li, Li Wan

**Affiliations:** ^1^ Department of Pain Management, The State Key Specialty in Pain Medicine, The Second Affiliated Hospital, Guangzhou Medical University, Guangzhou, China; ^2^ Sino-French Hoffmann Institute, School of Basic Medical Sciences, Guangzhou Medical University, Guangzhou, China; ^3^ Stem Cell Translational Medicine Center, The Second Affiliated Hospital, Guangzhou Medical University, Guangzhou, China

**Keywords:** DNFB-induced chronic itch, *Nlrp3* inflammasome, type 2 immunity, TRPA1, IL-18

## Abstract

**Background:**

The nod-like receptor family pyrin domain-containing 3 (NLRP3) has been implicated in various skin diseases. However, its role in mediating 2, 4-dinitrofluorobenzene (DNFB)-induced chronic itch remains unclear.

**Methods:**

Widetype (*WT*) and *Nlrp3* deletion (*Nlrp3^-/-^*)mice, the expression of transient receptor potential (TRP) ankyrin 1 (TRPA1) inhibitor or recombinant mice interleukin-18 (IL-18) were used to establish and evaluate the severity of DNFB-mediated chronic itch. Quantitative real-time PCR, western blotting, immunohistochemistry staining, immunofluorescence staining and enzyme-linked immunosorbent assay (ELISA) was used to examine the expression of NLRP3 inflammasome, type 2 immunity and receptors in dorsal root ganglion (DRG) neurons related with chronic itch. Flow cytometry was performed to quantify the frequency of type 2 immune cells.

**Results:**

This study revealed that the NLRP3 inflammasome was activated in the skin of DNFB-induced chronic itch mice. Surprisingly, the absence of Nlrp3 exacerbated itch behavior. In *Nlrp3^-/-^* mice, IL-18 expression was downregulated, whereas markers of type 2 immunity, such as IL-4 and thymic stromal lymphopoietin (TSLP), were significantly upregulated in the skin. Furthermore, TRPA1 and its colocalization with the IL-4 receptor were increased in the DRG. Inhibition of TRPA1 or administration of recombinant IL-18 significantly reduced DNFB-induced itch behavior in Nlrp3-/- mice. Recombinant IL-18 also decreased the expression of TRPA1, IL-4, and TSLP.

**Discussion:**

These findings suggested that the absence of Nlrp3 aggravated DNFB-induced chronic itch by exacerbating type 2 immunity in the skin and enhancing the IL-4/TSLP-TRPA1 axis, potentially driven by reduced IL-18 levels.

## 1 Introduction

Chronic itch is a highly prevalent disorder that occurs in various forms of dermatitis, including allergic contact dermatitis (ACD), atopic dermatitis (AD), and certain systemic and neuropathic disorders, with limited therapeutic options. Most chronic itch conditions are thought to arise from complex crosstalk between the immune and nervous systems (1, 2). Among these, type 2 immunity plays a crucial role in chronic pruritic inflammatory skin disease, mediated by interactions between skin barrier damage and abnormal immune responses (3). When keratinocytes are exposed to allergens, they release alarmins such as thymic stromal lymphopoietin (TSLP), which activate itch sensory neurons and initiate immune cells to release cytokines such as interleukin-4 (IL-4) and IL-13 (2–5). However, the role of the nucleotide-binding and oligomerization domain (nod)-like receptors (NLR) family pyrin domain containing 3 (NLRP3) in allergen-induced chronic itch remains unexplored.

NLRP3 is an intracellular sensor that detects a wide range of microbial and danger signals, triggering the assembly and activation of the NLRP3 inflammasome. Activation of the NLRP3 inflammasome has been implicated in the development of allergic skin inflammation or contact hypersensitivity (CHS), although the findings remain contradictory (6–9). In the skin of patients with AD, the expression of NLRP3 is reduced (10). Conversely, chronic AD-like inflammation, independent of NLRP3 inflammasome activation, relies on IL-1β and IL-1 receptor 1 (IL-1R1) signaling (11). A recent study reported that an NLRP3 inflammasome inhibitor enhances imiquimod-induced chronic itch through the NLRP3/Caspase-1/IL-1β axis (12). However, no study has yet investigated the role of NLRP3 in DNFB-induced chronic itch using *Nlrp3* ^-/-^ mice.

Therefore, we aimed to investigate the role of NLRP3 in DNFB-induced chronic itch using *Nlrp3* knockout mice and elucidate the mechanisms underpinning skin immunity-neuron interactions.

## 2 Materials and methods

### 2.1 Reagents, drugs, and antibodies

1-Fluoro-2, 4-dinitrobenzene (DNFB) was acquired from Xiya Reagents (China). The BCA protein assay kit (23227) was from Thermo Fisher Scientific, USA. An Anti-Mouse IL-4 ELISA Kit (VAL603) and the expression of transient receptor potential (TRP) ankyrin 1 (TRPA1) antibody (NB110-40763) were obtained from Novus Biologicals, USA. Antibodies (Abs) including anti-TSLP (ab188766), IL-18 (ab71495), TRP vanilloid 1 (TRPV1) (ab31895), Interleukin-4 receptor alpha (IL-4RA) (ab203398), goat anti-rat conjugated rhodamine 555, rabbit anti-goat conjugated FITC, and CD45-APC (104) were from Abcam, Britain. Liberase and DNase were from Roche, Switzerland. Various Abs for flow cytometry and reagents, including anti-CD45-APC-CY7 (30-F11), CD11b (M1/70), SiglecF (E50-2440), FceR1α-APC-eFluor 780 (MAR-1), CD117-Per-cy5.5 (2B8), CD127-KO525 (SB/19), IL-33 receptor (ST2)-BV421 (U29-93), CD3ϵ-Alexa 488 (145-2C11), CD4-BV510 (RM4-5), Anti-Mouse IgE ELISA Kit (555248), Fc-blocking reagent (CD16/CD32 Pure 2.4G2, 553142), and Fixable Viability Stain 570 (564995), were ordered from BD Biosciences (San Jose, CA). Additionally, biotin-labeled anti-mouse CD3 (17A2), CD4 (GK1.5), CD8a (53-6.7), CD11c (N418), NK1.1 (PK136), CD19 (6D5), Gr1(RB6-8C5), FcϵR1α (1-Mar), CD5 (53-7.3), F4/80 (BM8), anti-mouse IL-4-PerCP-eFluor 710 (11B11), and Zombie Green™ Fixable Viability Kit (423111) were from BioLegend, USA. Anti-IL-1β (AF-401-NA) and Anti-Mouse IL-1β ELISA Kit (DY-404) were from the R&D system, USA. Anti-NLRP3 (15101), Apoptosis-associated speck-like protein containing a caspase recruitment domain (ASC) (67824), Ly6G (32469), and rabbit mAb IgG (DA1E) as isotype control were obtained from Cell Signaling Technology, USA. Avidin-conjugated FITC (189727) was purchased from Sigma-Aldrich, USA. Anti-GAPDH (ABclona, AC002, China) and β-actin (Ray antibody, RM2001, China) were also utilized. The TRPA1 antagonist (HC030031) was from ApexBio, (USA). Recombinant mouse IL-18 (rmIL-18) was purchased from Medical and Biological Laboratories CO., LTD. (Nagoya, Japan, B004-2). Donkey anti-Rabbit IgG Alexa Fluor 488 and Alexa Fluor™ 594 Tyramide reagent were purchased from Life Technologies Invitrogen, USA.

### 2.2 Mice and ethics statement and treatment

Seven to 12-week-old male mice were used for the experiments. C57BL/6 wild-type (*WT*) mice were purchased from the Guangdong Medical Laboratory Animal Center, and *Nlrp3*-knockout mice (*Nlrp3^-/-^*, Stock No: 021302) were purchased from Jackson Laboratory, USA. All mice were housed in groups of five per cage under controlled conditions in sound-proof, humidity-controlled chambers with a 12-h light/dark cycle. Food and water were provided *ad libitum*. All animal experiments were performed following the guidelines of the National Institutes of Health and the International Association for the Study of Pain. The experimental protocols were approved by the Institutional Animal Care and Use Committee at Guangzhou Medical University (Approval No. GY2019-007).

Chronic itch was induced by hapten 1-fluoro-2, 4-dinitrobenzene (DNFB, Xiya Reagents, China). The mice are randomly assigned to the DNFB group (DNFB, sensitized and challenged with DNFB) or negative control group (NC, sensitized and challenged with acetone). The hair on the abdomen and the nape of the neck was shaved on day -7. Male *WT* and *Nlrp3* ^-/-^ mice were sensitized on day -5 by applying 100μl of 0.15% DNFB to the shaved abdominal skin. The mice were then challenged on days 0, 2, 4, and 6 by painting 50μl of 0.15% DNFB onto the shaved nape of the neck (13). Scratching behavior was video-recorded for 1 h on days 1, 3, 5, and 7. A scratching bout was defined as the act of lifting the hind limb to the painted (nape) or injection site, followed by returning the limb to the floor or to the mouth, regardless of the number of scratching strokes within the bout. The number of scratching bouts was counted in a blinded manner.

The role of TRPA1 in DNFB-mediated chronic itch in *Nlrp3* ^-/-^ male mice was assessed by intrathecal injection of a selective TRPA1 antagonist, HC-030031 (100 mg/kg,100μl in 20% SEB-β-CD, ApexBio, USA.), or a vehicle control, administrated 30 mins after each challenge (five mice per group). To assess the contribution of IL-18 in DNFB-mediated chronic itch in *Nlrp3* ^-/-^ male mice, intradermal (i.d.) injections of recombinant mouse IL-18 (rmIL-18, 2 µg in100μl PBS) were administrated daily to the shaved back on days 1, 3, 5, and 7. Scratching behavior was video-recorded for 1 h before and 1 h after each injection.

### 2.3 RNA isolation and reverse transcript quantitative PCR

Total RNA from fresh mouse nape skin and DRGs was extracted using the Trizol reagent (Invitrogen, 15596026, USA). Genomic DNA contamination was removed, and mRNA was reverse-transcripted using the PrimeScript™ RT reagent kit with gDNA Eraser (RR047Q, Japan). Quantitative real-time PCR (qPCR) was performed with the SYBR^®^ Premix Ex Taq™ II kit (RR037A, Japan) on a Bio-Rad C1000 Thermal Cycler, following the manufacturer’s instructions. The forward and reverse primer sequences are listed in **Supplementary Table S1**. The relative mRNA expression levels of individual genes were normalized to GAPDH and calculated using the 2^-ΔΔCt^ method. Fold changes were determined with the level of *Gapdh* in the NC (*WT*) or DNFB (*WT*) group set as 1. Data points with Z-scores greater than 3 or less than -3 were excluded from the analysis.

### 2.4 Enzyme-linked immunosorbent assay

Mouse serum was harvested to measure IgE levels, while lysate from the shaved skin area were prepared to measure IL-1β and IL-4 levels according to the instructions of their respective ELISA kits. Skin tissue was homogenized in a buffer containing 0.5% acetyltrimethyl ammonium bromide and 2% fetal bovine serum (FBS) in PBS. The homogenate was centrifuged at 13,000g for 15 min at 4°C. The resulting supernatant or skin lysate was collected and normalized with the BCA Protein Assay Kit.

### 2.5 Immunohistochemistry staining

OCT-embedded skin sections were permeabilized with PBS for 10 min and pretreated with 3% H_2_O_2_ for 10 min to eliminate peroxidase activity. This was followed by treatment with 0.3% Triton X-100 for 10 min. The sections were blocked with 10% bovine serum albumin (BSA) for 1 hour at room temperature before being incubated with a rabbit anti-mouse TSLP primary antibody or isotype control-rabbit mAb IgG (DA1E) overnight at 4℃. Then, the slides were incubated with an HRP-conjugated goat anti-rabbit secondary antibody for 1 hour at room temperature. The stained sections were visualized and captured using an optical microscope equipped with a camera (Olympus, Tokyo, Japan). The semi-quantitative analysis of TSLP expression was determined by image J software and the modified H-score method (14).

### 2.6 Flow cytometry analysis

Fresh mice nape skin samples were cut, separated with forceps, and digested in a solution containing 0.25mg/ml liberase and 0.4 mg/ml DNase for 120 minutes at 37℃. The resulting cells were filtered through a 100μm cell strainer. Red blood cells were lysed with ammonium chloride (NH_4_Cl) for 10 min. The single-cell suspension was then incubated with an Fc-blocking reagent before being stained with the indicated fluorescence-conjugated anti-mouse antibodies. Dead cells were excluded using either a Zombie Green™ Fixable Viability Kit or the Dead and Fixable Viability Stain 570. Stained cells were acquired using a CytoFLEX flow cytometer (Beckman Coulter), and data analysis was performed using FlowJo software (Ashland, OR). Cell populations were identified as follows: Eosinophils (Eos) were gated as CD45^+^CD11b^+^SiglecF^+^, basophils (BSs) as CD45^+^CD11b^+^IgER^+^CD117^–^, mast cells (MCs) as CD45^+^CD11b^+^IgER^+^CD117^+^, ILC2 as CD45^+^Lin^-^CD127^+^GATA3^+^, and Th2 cells as CD45^+^CD3^+^CD4^+^IL-4^+^. The lineage (Lin) markers used included anti-CD3, CD4, CD8a, CD11c, NK-1.1, CD19, TER-119, Ly6G/Ly6C, FcϵRIα, and F4/80.

### 2.7 Western blotting

The skin from the back of the neck was cut and dissolved in RIPA lysis buffer supplemented with a complete protease inhibitor cocktail (Beyotime, China). The tissues were homogenized with an electric homogenizer (Retsch MM400, German). The homogenate was centrifuged, and the supernatant was restained. Protein concentrations were measured using a BCA protein assay kit (Thermo Fisher Scientific, USA), and the protein samples were diluted with loading buffer and boiled for 5 min. Equal quantities of total protein lysates were separated on 10% sodium dodecyl sulfate-polyacrylamide gel electrophoresis (SDS-PAGE) for denatured samples or on nondenaturing polyacrylamide gels (without SDS) for ASC oligomer detection. Proteins were then transferred to PVDF membranes. The membranes were incubated with the indicated primary and secondary antibodies. Protein signals were visualized using an ECL Western blotting substrate (Solarbio) and detected with a Fusion SoloS imaging system (VILBER LOURMAT). The intensity of target protein bands was semi-quantified by ImageJ software and normalized to the corresponding input control bands (GAPDH or β-tubulin). Fold changes were calculated with the control set as 1.

### 2.8 Immunofluorescence staining

OCT-embedded skin or DRG sections from WT or *Nlrp3* ^-/-^ mice, with or without DNFB-mediated chronic itch, were incubated overnight at 4℃ with primary antibodies against ASC, NLRP3, IL-18, IL-1β, Ly6G, TRPA1, TRPV1, or IL-4RA. For the isotype control, sections were incubated with rabbit mAb IgG (DA1E) or 5% BSA instead of primary Abs. Afterward, sections were incubated with Alexa Fluor-488- or Alexa Fluor-594-conjugated anti-rabbit or anti-rat second Abs for 1 h at room temperature in the dark. To assess the frequency of mast cells, skin sections were directly incubated with avidin-conjugated FITC. To ascertain the ratio of cells with double positive IF staining in the DRG, sections were incubated with HRP-conjugated goat anti-rabbit second Ab following anti-IL-4RA staining, and the signals were developed with Alexa Fluor™ 594 Tyramide reagent. IL-4RA antibodies were then removed with antibody eluent (Absin, China), followed by incubation with TRPA1 antibodies. Cell nuclei were counterstained with DAPI for 10 min. Finally, slides were mounted with an antifade medium (Vectashield, Vector Laboratories). Images were captured using a Zeiss LSM Pascal Axiovert confocal microscope (Carl Zeiss). The number of single-positive cells (avidin, ASC, NLRP3, Ly6G, IL-1β, TRPA1, TRPV1, and IL-4RA) and double-positive cells (Ly6G/IL-1β or TRPA1/IL-4RA) in each field at 200×magnification was quantified using Image J software. Five random images were selected from each section for analysis. The staining intensity of these images was quantified with a preselected threshold in Image J.

### 2.9 Hematoxylin and eosin staining

The painted nape skin of mice was collected, fixed in 4% paraformaldehyde (PFA), and sequentially processed in 5%, 15%, and 30% sucrose solutions. The tissues were embedded in optimal cutting temperature (OCT) medium (SAKURA) and sectioned into 6μm using a cryostat (Thermo Scientific NX50). The sections were stained with H&E, and epidermal thickness was measured at three specific locations (left, center, and right) in each field under 200×magnification using Image J software. The number of infiltrated immune cells per field (magnification×200) in each section was assessed in a blinded manner. For each section, five random images were selected for analysis.

### 2.10 Statistical analysis

Data were presented as the mean ± standard error of the mean (SEM) from the indicated number of replicates or experiments. Statistical analyses, including correlation analysis, were performed using Prism 7 (V7.0d, GraphPad, San Diego, CA). Differences between two groups were analyzed using an unpaired *t*-test, while multiple comparisons were analyzed using one-way or two-way analysis of variance (ANOVA), followed by Bonferroni *post hoc* tests. A *P*-value of ≤ 0.05 was considered statistically significant.

## 3 Results

### 3.1 The NLRP3 inflammasome was activated in the skin of mice with DNFB-induced chronic itch

**Figure 1:**
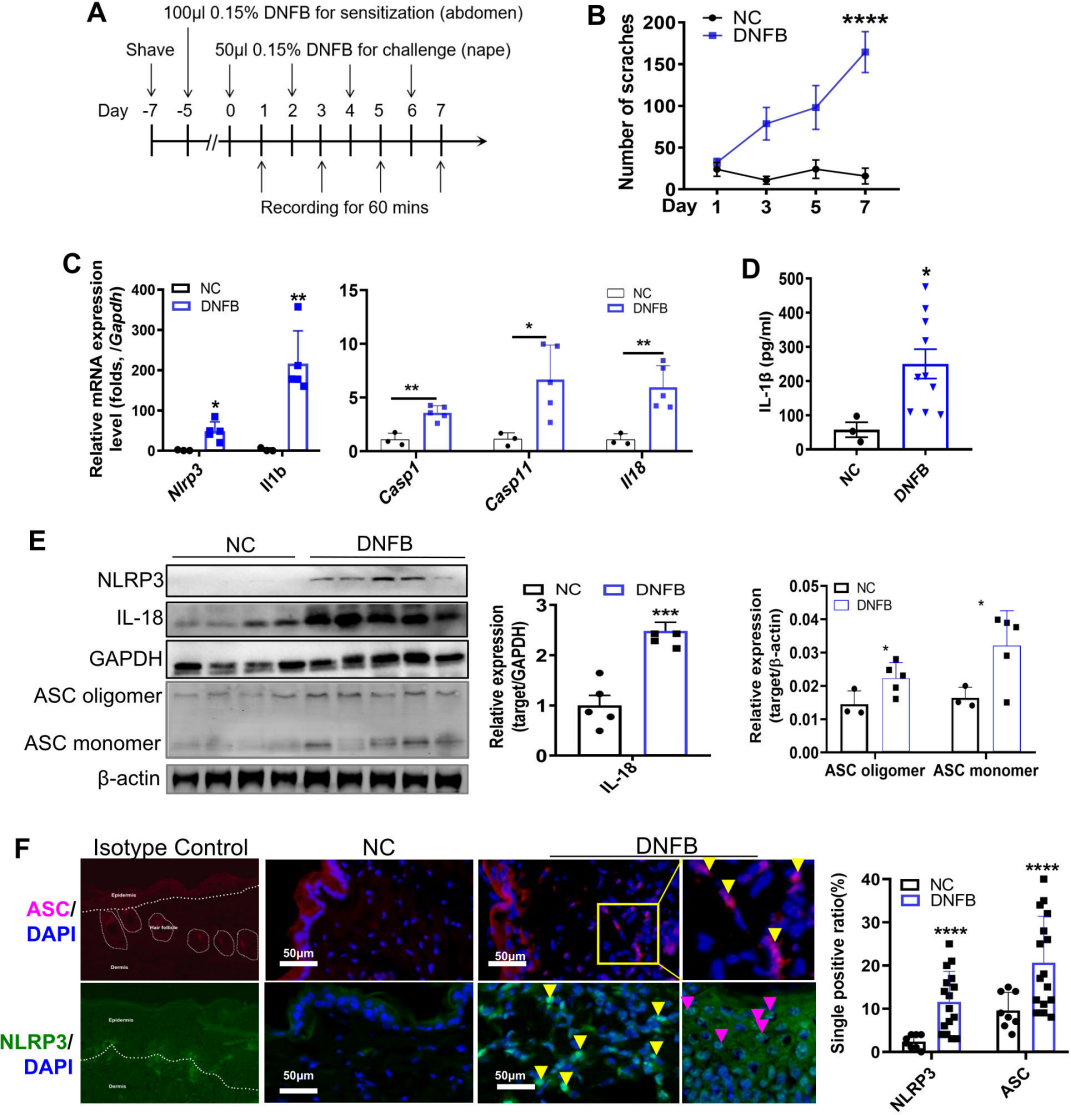
NLRP3 inflammasome was activated in the skin of mice with DNFB-induced chronic itch. **(A)** Flowchart illustrating the establishment of the DNFB-induced chronic itch model. **(B)** The number of scratches within 1 hr was video recorded in normal controls (group NC) and mice with DNFB-induced chronic itch (group DNFB). **(C)** Relative RNA expression levels of *Nlrp3*, *Il1b*, *Casp1*, *Casp11*, and *Il18* in the skin of indicated mice were detected by RT-qPCR. **(D)** IL-1β concentration in the skin lysate was analyzed by ELISA. **(E)** Protein expression levels of NLRP3, IL-18, and ASC (both oligomer and monomer forms) were detected by Western blotting (WB). Representative WB images are shown on the left, and semi-quantitative analysis is presented on the right. **(F)** Expression of NLRP3 and ASC in skin sections from mice with or without DNFB challenge was visualized by IF staining. Representative IF photographs (400× magnification) are shown on the left, and the percentage of NLRP3 or ASC single positive cells is shown on the right. For each experiment, the sample size was n=5 (NC group) or 5-7 (DNFB), and all experiments were conducted in duplicate. Quantitative data are shown as mean ± SEM. Statistical significance was determined using an unpaired *t*-test, with *P<0.05, **P<0.01, ***P<0.001, and ****P<0.0001.

A DNFB-induced chronic itch model was established in C57BL/6 mice as previously described (7) (**Figure 1A**). Mice subjected to DNFB-induced chronic itch (referred to as “DNFB”) exhibited persistent scratching behavior following DNFB treatment, with the number of scratches progressively increasing compared to the control mice (referred to as “NC”; **Figure 1B**). The components of the NLRP3 inflammasome and its downstream cytokine, IL-1β, have been reported to play important roles in CHS and AD (8–11, 15, 16). However, the role of NLRP3 in DNFB-induced chronic itch remains poorly understood. Upon NLRP3 inflammasome activation, NLRP3 recruits ASC, forming ASC specks that cluster pro-caspase1, facilitating its cleavage into the active p20/p10 heterotetramer. This activated complex subsequently cleaves pro-IL-1β and pro-IL-18 into their mature forms, IL-1β (p17) and IL-18 (17). In our study, we found a significant upregulation in the RNA expression levels of *Nlrp3* and *Il1b* in the skin of DNFB-treated mice, showing approximately 50- and 200-fold increases, respectively, compared to the control mice. Additionally, the transcription levels of *Caspase1* (*Casp1*), *Caspase11* (*Casp11*) and *Il18* were also significantly increased (**Figure 1C**). Furthermore, the protein expression levels of IL-1β (**Figure 1D**), NLRP3, IL-18, ASC oligomer, and monomer from skin lysate (**Figure 1E**), and Caspase1 and Caspase11 (**Supplementary Figure S1**), which are markers of canonical and non-canonical NLRP3 inflammasome activation, were significantly increased. To further detect the precise localization of NLRP3 inflammasome activation in the scratched skin, IF staining for ASC and NLRP3 was performed. The percentages of ASC- and NLRP3-positive cells increased in the epidermis during the DNFB challenge (**Figure 1F**). Notably, NLRP3 and ASC-positive staining were prominently observed in the cytoplasm of infiltrating immune cells in the dermis (**Figure 1F**, yellow arrows) and in the epidermis (purple arrows) of DNFB- challenged mice. These findings indicate that NLRP3 inflammasome activation was evident in the skin of mice with DNFB-induced chronic itch, particularly in the cells of the dermis layer, as demonstrated by the apparent NLRP3 and ASC-positive staining.

### 3.2 *Nlrp3* genetic deletion exacerbated DNFB-induced chronic itch in mice

**Figure 2:**
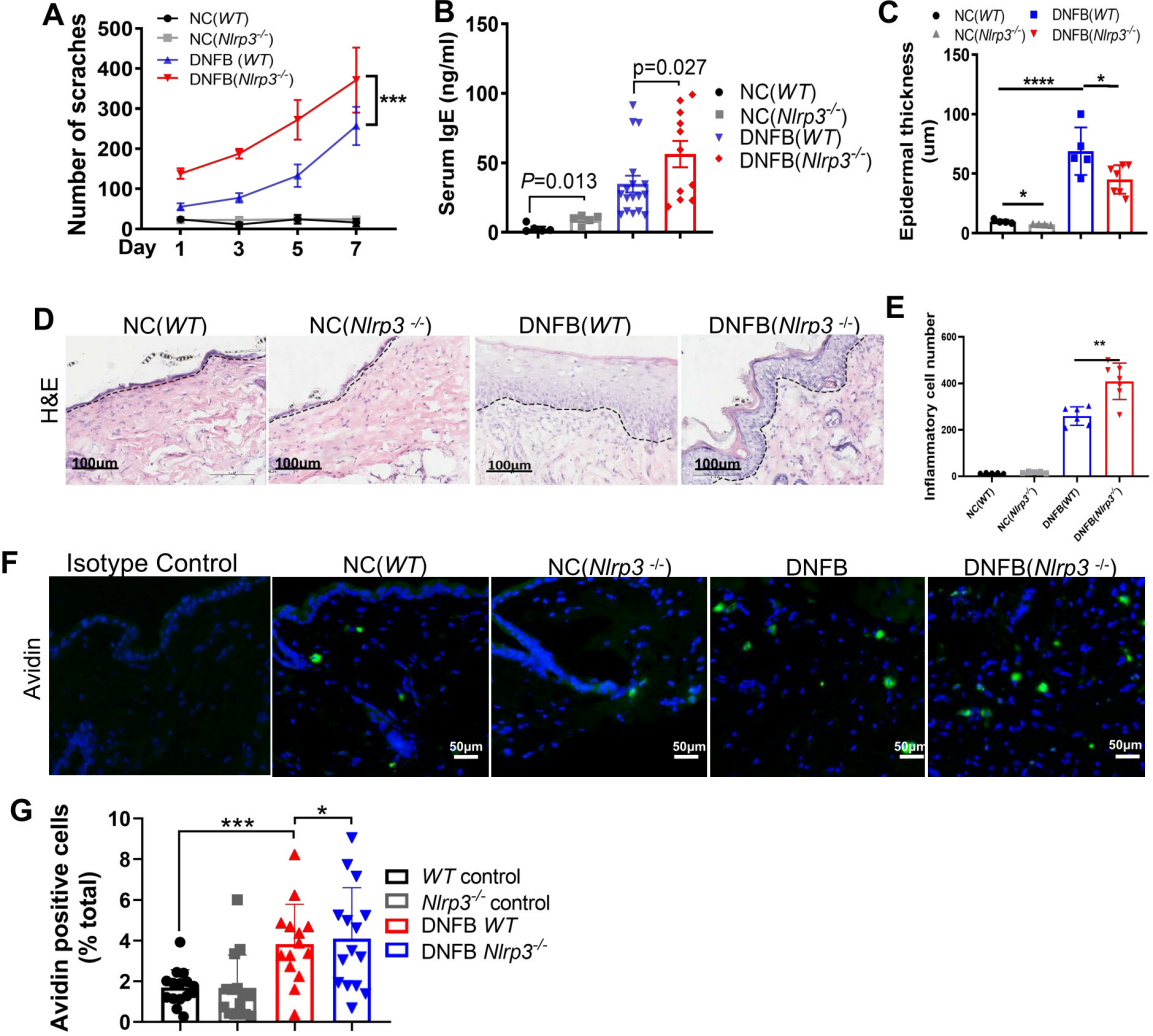
*Nlrp3* genetic deletion exacerbated DNFB-induced chronic itch. **(A)** The number of scratching bouts within 1 hour was recorded via video monitoring in *WT* and *Nlrp3^-/-^* mice subjected to DNFB-induced chronic itch. **(B)** Serum IgE concentrations in *WT* mice or *Nlrp3^-/-^* mice with DNFB-induced chronic itch were detected using an IgE ELISA kit. **(C–E)** Epidermal thickness and inflammatory cell infiltration in the dermis of the indicated mice were analyzed through H&E staining. **(F, G)** Avidin staining (representative photographs shown in **(F)**) and quantitative analysis **(G)** of avidin-positive cells or the ratio of positive cells to total cells in 200× magnification fields of skin sections were performed. The number of mice in the NC group was 5, and the DNFB (*WT*) or DNFB (*Nlrp3* ^-/-^) groups each included eight mice, with experiments repeated twice. All quantitative data are shown as mean ± SEM. *P<0.05, **P<0.01, ***P<0.001, ****P<0.0001. Two-way ANOVA was used for **(A)**, *t*-test was used for **(B)**, and one-way ANOVA was applied for **(C, E, G)**.

To further investigate the role of NLRP3 in DNFB-induced chronic itch, we used *Nlrp3^-/-^* mice to establish a chronic itch model. Compared to *WT* mice, *Nlrp3^-/-^* mice subjected to DNFB-induced chronic itch exhibited a significantly increased number of scratching behaviors (**Figure 2A**). Serum IgE levels (**Figure 2B**), the number of infiltrating avidin-positive mast cells (**Figures 2F, G**), and the infiltration of immune cells in the dermis were all significantly elevated (**Figures 2D, E**). Additionally, epidermal thickness was notably reduced (**Figures 2C, D**). Our results demonstrated that DNFB challenge induces persistent itching, impairs the skin barrier, and enhances inflammatory cell infiltration into the dermis of mice, closely mimicking the key characteristics of AD. Collectively, these findings indicate that the genetic deletion of *Nlrp3* aggravates DNFB-induced chronic itch.

### 3.3 *Nlrp3* genetic deletion diminished IL-18 but not IL-1β in DNFB-induced mice

**Figure 3:**
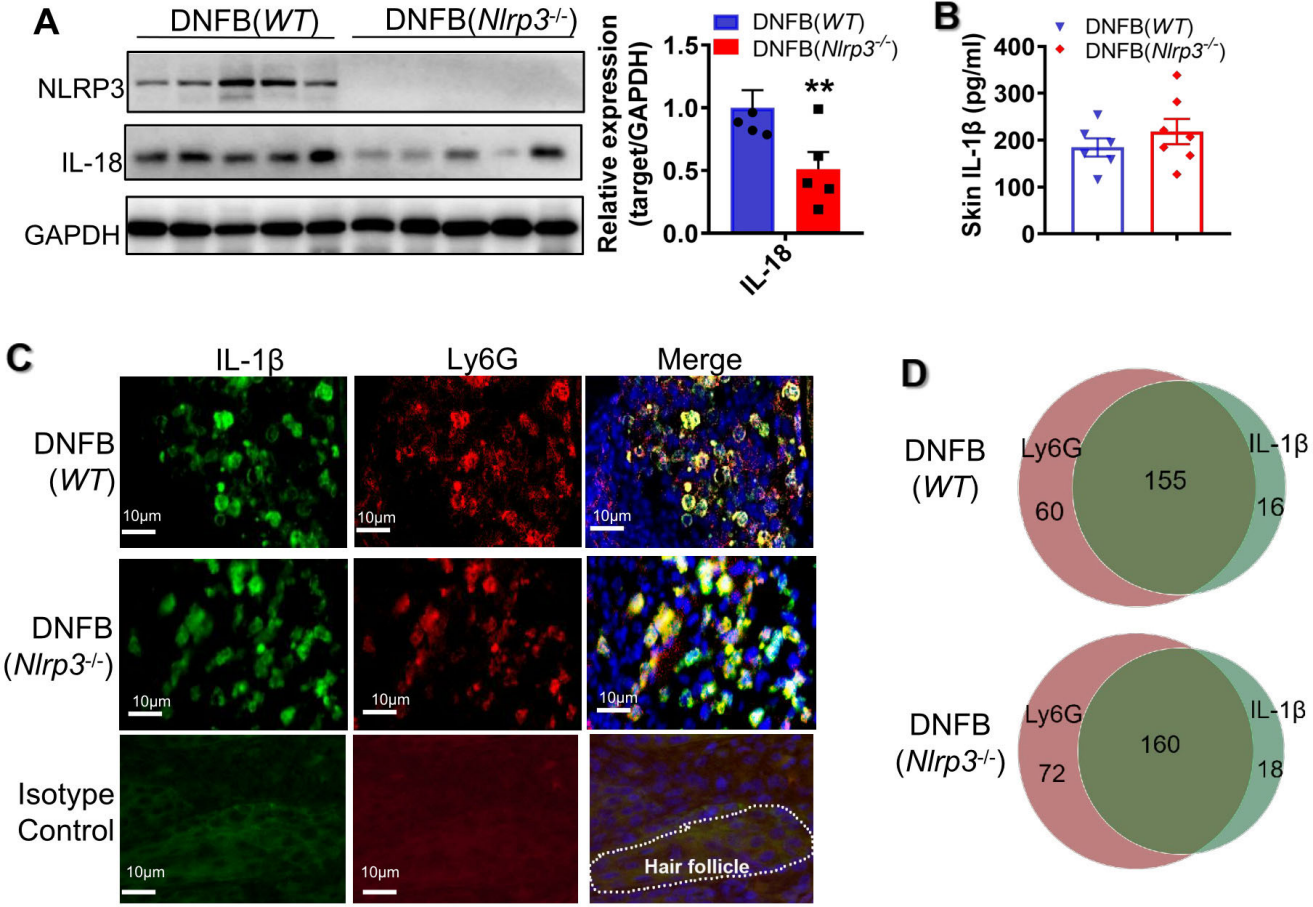
*Nlrp3* deletion diminished IL-18 but not IL-1β in DNFB-induced mice. **(A)** The protein expression levels of NLRP3 and IL-18 in the skin lysates of DNFB-challenged *WT* and *Nlrp3^-/-^* mice were detected by WB. The representative bands are shown on the left, and the semi-quantitative analysis is presented on the right. **(B)** Representative photographs of IL-18 positive IF staining in mice skin sections (400×) ware are shown on the left, demonstrating a significant decrease in IL-18 positive cells due to *Nlrp3* deletion. **(C)** The IL-1β concentration in skin lysates of the indicated mice was analyzed by ELISA. **(D)** Representative photographs of IL-1β (green) or Ly6G (red) single positive and their merged (yellow) IF staining in mice skin sections are shown on the left (400×). Quantification of IL-1β, Ly6G single-positive, and double-positive cells per field (100×) is shown on the right. All quantitative data are presented as mean ± SEM. **P<0.01, unpaired *t*-test.

Given that *Nlrp3*-knockout (KO) mice exhibited increased scratching behavior in a DNFB-induced chronic itch model, we further investigated whether IL-18 or IL-1β plays a vital role in NLRP3 inflammasome activation. In DNFB-challenged *Nlrp3*-KO mice, the protein expression level of IL-18 in the skin was significantly decreased (**Figures 3A, B**), whereas the cleaved p20/p10 heterotetramer of caspase1 and the cleaved p25 heterotetramer of caspase11 showed no significant changes (**Supplementary Figure S2**). Furthermore, the concentration of IL-1β in the skin lysate remained unchanged (**Figure 3C**). It has been reported that IL-1β can be produced via NLRP3 inflammasome-independent mechanism, with neutrophil-derived proteases playing a role in processing pro-IL-1β into its mature form (18). Consistent with this, our findings showed that the majority of IL-1β was localized within neutrophils in the dermal layer of both *WT* mice and *Nlrp3* ^-/-^ mice with DNFB-induced chronic itch (**Figure 3D**). In line with previous studies (18, 19), our results suggested that the enhanced IL-1β expression in the skin is partly independent of NLRP3 inflammasome activation. Thus, these findings indicate that in DNFB-induced chronic itch in mice, the activation of NLRP3 inflammasome in the skin primarily relies on IL-18 rather than IL-1β.

### 3.4 *Nlrp3* genetic deletion promoted type 2 immunity in DNFB-induced mice

As previously reported, the recruitment of immune cells and the release of cytokines such as IL-4, IL-13, IL-33, IL-31, IFNγ, and TSLP are hallmarks of AD (20). In our study, we observed that the transcriptional levels of type 2 cytokines *Il4* and *Il13* were significantly increased in the skin of DNFB-induced chronic itch, whereas *Il33* and *Il10* levels remained unchanged (**Supplementary Figure S3A**). The levels of *Il5* and *Il25* were undetectable due to being below the detection threshold (data not shown). Consistently, ELISA analysis showed a significant increase in the concentration of IL-4 in skin lysates (**Supplementary Figure S3B**). Moreover, the protein expression of TSLP in the epidermal layer of mice with chronic itch was significantly increased (**Supplementary Figure S3C**). Furthermore, several type 2 immune cells, including Eos, MCs, and BSs, were significantly increased in the skin of DNFB-induced chronic itch mice. However, the numbers of ILC2s were dramatically decreased, and Th2 cells did not show significant changes (**Supplementary Figure S3D**).

**Figure 4:**
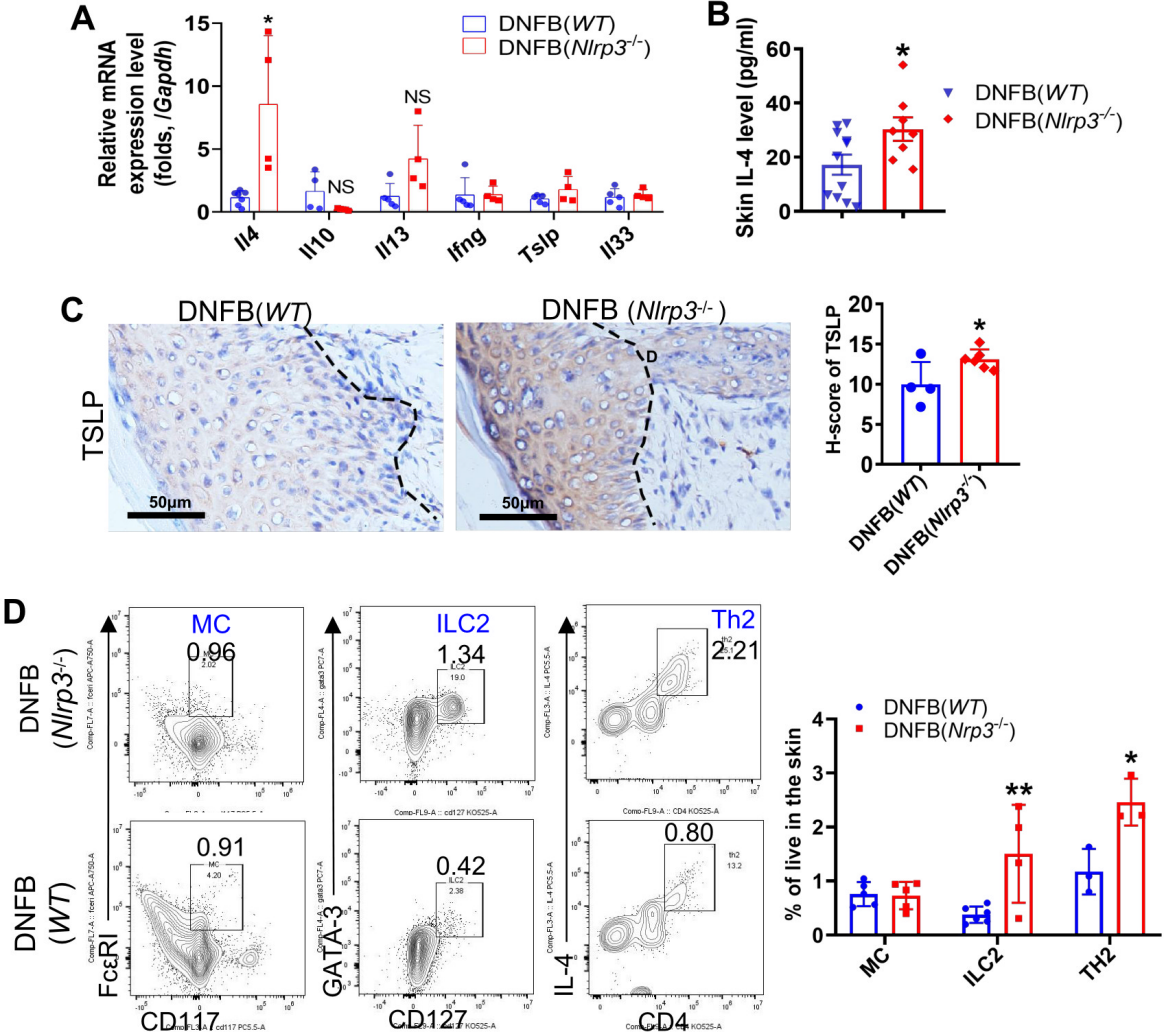
Knockout of *Nlrp3* enhanced ILC2, Th2 cells and type 2 immunity in the DNFB-induced chronic itch mouse skin. **(A)** The relative mRNA expression levels of *Il4*, *Il10*, *Il13*, *Ifnγ*, *Tslp*, and *Il33* in the skin of the indicated mice were detected by RT-qPCR. **(B)** The IL-4 concentration in the neck skin lysates of the indicated mice was analyzed by ELISA. **(C)** The expression level of TSLP in neck skin was detected by IHC. Representative photographs are shown on the left, with semi-quantitative analysis shown as H-scores on the right. **(D)** Frequencies of the indicated cell types isolated from the neck skin of *WT* mice or *Nlrp3* ^-/-^ mice with DNFB were analyzed by flow cytometry and FlowJo software. Representative gating plots are shown on the left and their quantifications are displayed on the right. The sample size for the DNFB (*WT*) or DNFB (*Nlrp3* ^-/-^) groups was eight. All quantitative data are shown as mean ± SEM. Statistical significance was determined by unpaired t-test: *P<0.05, **P<0.01.

To further investigate the effect of NLRP3 deficiency on the exacerbation of chronic itch, we performed RT-qPCR to detect the expression of cytokines, including IL-4, IL-13, IL-33, IL-31, IFNγ, and TSLP, and other molecules such as KLKs and cathepsin S. Among these, only *Il4* showed a significant elevation, with about an 8.5-fold increase (**Figure 4A**). In contrast, the level of *Il5* was undetectable (data not shown). We further quantified the concentration of IL-4 in skin lysates using ELISA (**Figure 4B**) and assessed TSLP protein expression by IHC staining (**Figure 4C**). Both IL-4 and TSLP were significantly increased in the skin of DNFB-challenged *Nlrp3-*knockout mice (**Figures 4B, C**). Additionally, flow cytometry and FlowJo analysis revealed a significant increase in the frequency of ILC2 and Th2 cells, while MC frequency remained unchanged (**Figure 4D**). Based on our findings, we conclude that IL-4, TSLP, and the increased presence of ILC2 and Th2 cells contribute to type 2 immunity, which plays a critical role in aggravating DNFB-induced chronic itch in the skin of *Nlrp3* ^-/-^ mice.

### 3.5 TRPA1 in the DRG was associated with a high frequency of DNFB-induced chronic itch in *Nlrp3^-/-^* mice

**Figure 5:**
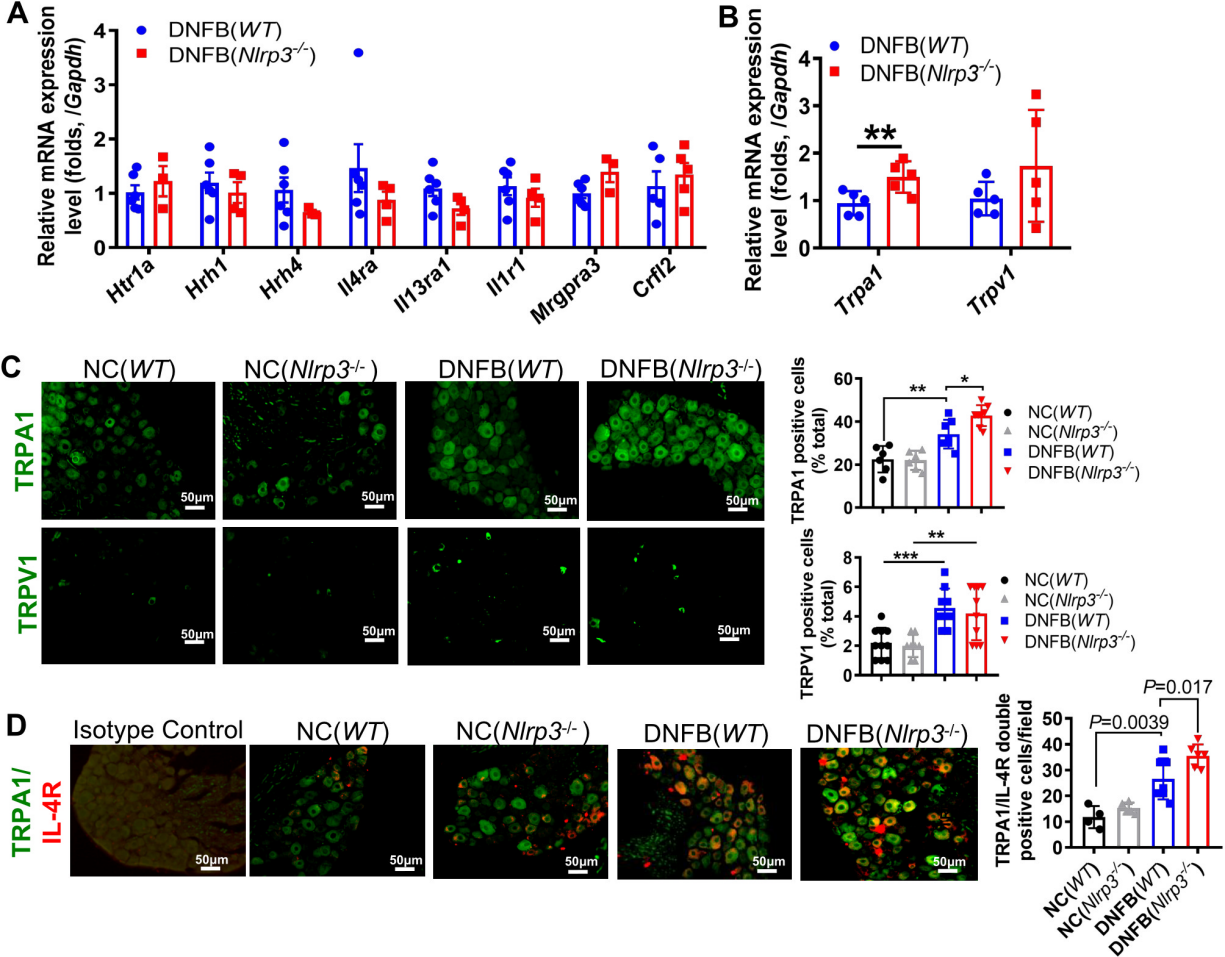
TRPA1 neurons were involved in severe DNFB-induced chronic itch of *Nlrp3^-/-^* mice. **(A)** RNA expression levels of receptors related to chronic itch in the DRG were analyzed by RT-qPCR. **(B)** RNA expression levels of *Trpa1* and *Trpv1* in the DRG of DNFB-challenged mice. **(C)** IF staining of TRPA1 and TRPV1 in the DRG. Representative photographs are shown on the left, and the quantitative analyses are presented on the right, illustrating the TRPA1 and TRPV1 positive ratios per 100 cells in the DRG sections of mice. **(D)** IF staining of TRPA1 and IL-4R in the DRG. Representative photographs are shown on the left, and the quantitative analyses are shown on the right, illustrating the average count of TRPA1 and IL-4R double-positive cells per field (400×) in the DRG sections of mice. The sample size in the NC group was five and in DNFB (*WT*) or DNFB (*Nlrp3* ^-/-^) group, it was eight. All quantitative data are shown as mean ± SEM. Statistical significance is indicated as follows: *P<0.05, **P<0.01, ***P<0.001. One-way ANOVA was used for the analyses in **(C, D)**, while an unpaired *t*-test was applied in **(A, B)**.

The activation of ion channels, including transient receptor potential (TRP) cation channels, particularly TRP ankyrin 1 (TRPA1) and TRP vanilloid 1 (TRPV1), and voltage-gated sodium channels in the DRG, is essential for the transduction of chronic itch signals (21). To ascertain the role of itch sensory neuron receptors in DNFB-induced chronic itch, we examined the RNA expression levels of pruritogens’ receptors, TRPA1, and TRPV1, using RT-qPCR. The mRNA expression levels of 5-HT receptors (*5Hrta1*, *H1r*, and *H4r*), IL-1 receptor 1 (*Il1r1*), IL-4 receptor alpha (*Il4ra*), *Mrgpa3*, and TSLP receptor (*Crfl2*) showed no significant changes in *Nlrp3*-deficient mice (**Figure 5A**). Similarly, the transcriptional expression of *Trpv1* did not differ significantly, whereas *Trpa1* was notably upregulated (**Figure 5B**). Correspondingly, the protein level of TRPA1, but not TRPV1, was significantly increased in the DRG neurons of DNFB-challenged *Nlrp3^-/-^* mice (**Figure 5C**).

During *Nlrp3* deletion, IL-4 and TSLP levels were elevated in the skin, accompanied by enhanced TRPA1 expression in the DRG. Previous studies have shown that TSLP secreted by keratinocytes can bind to the TSLP receptor in the DRG, directly affecting a subset of TRPA1-positive sensory neurons and triggering robust itch behaviors (22). In our study, nearly all IL-4RA positive cells were co-localized with TRPA1 positive neurons, and the count of double-positive cells per field significantly increased in DNFB-challenged *Nlrp3* ^-/-^ mice (**Figure 5D**). These findings suggest that IL-4 may bind to IL-4RA, subsequently activating TRPA1 on the same DRG neuron, thereby exacerbating scratching behavior in the absence of NLRP3.

### 3.6 IL-18 mediating the IL-4/TSLP-TRPA1 axis was associated with aggravated DNFB-induced chronic itch in *Nlrp3* ^-/-^ mice

**Figure 6:**
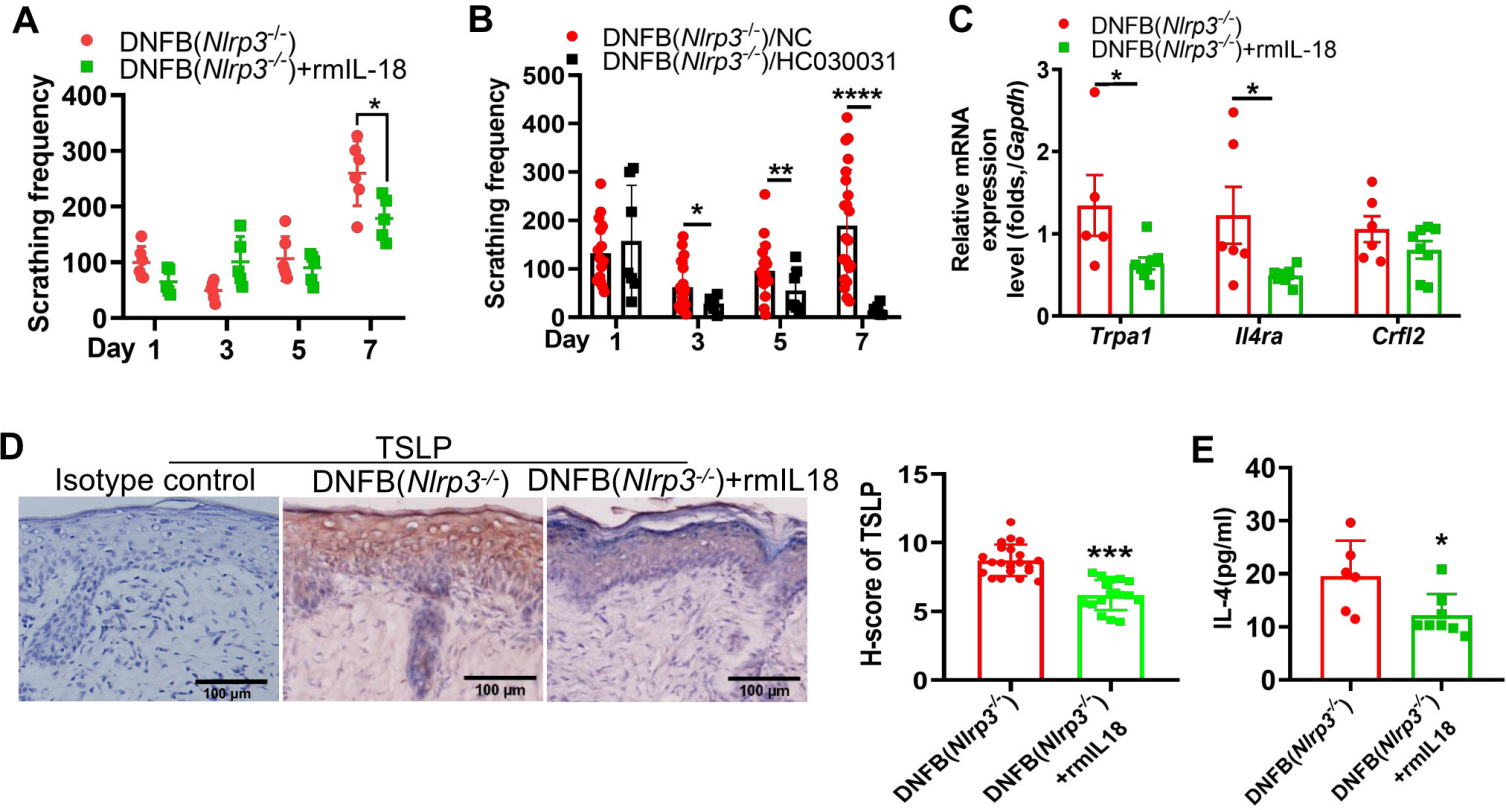
IL-18 was associated with the severity of DNFB-induced chronic itch via regulating type 2 immunity and TRPA1 in *Nlrp3* ^-/-^ mice. **(A)** Scratching frequency of DNFB-challenged *Nlrp3* ^-/-^ mice with or without rmIL-18 treatment. **(B)** Scratching frequency in DNFB-challenged *Nlrp3* ^-/-^ mice treated with or without TRPA1 antagonist (HC030031). **(C)** RNA expression levels of *Trpa1* in the DRG were analyzed by RT-qPCR. **(D)** TSLP expression in the neck skin of DNFB-challenged *Nlrp3* ^-/-^ mice, with or without rmIL-18 treatment, was detected by IHC. Representative photographs are shown on the left, with semi-quantitative analysis (H-score) on the right. **(E)** IL-4 concentrations in the neck skin lysate of DNFB-challenged *Nlrp3* ^-/-^ mice, with or without rmIL-18 treatment, were measured by ELISA. All quantitative data are presented as mean ± SEM. Statistical significance is indicated as follows: *P<0.05, **P<0.01, ***P<0.001, ****P<0.0001. A two-way ANOVA was used for **(A)**, and an unpaired *t*-test was applied for **(B–E)**.

As previously demonstrated, the DNFB-treated *Nlrp3* ^-/-^ mice exhibited decreased IL-18 levels alongside upregulated type 2 immune responses in the skin, accompanied by increased IL-4RA expression on TRPA1-positive neurons in the DRG. Correlation analysis revealed a negative association between DNFB-induced chronic itch frequency and IL-18 protein levels (**Supplementary Figure S5**). To further validate this relationship, recombinant mouse IL-18 (rmIL-18) was intradermally injected into *Nlrp3* ^-/-^ mice. Following the 4^th^ DNFB challenge, the number of scratching events was significantly reduced (**Figure 6A**). Additionally, intrathecal administration of HC030031, a TRPA1 antagonist, in the DNFB-treated *Nlrp3* ^-/-^ mice resulted in a significant decrease in itch behavior (**Figure 6B**). Protein levels of TSLP were significantly reduced (**Figure 6D**), though TSLP receptor RNA expression remained unchanged (**Figure 6C**). Furthermore, recombinant mouse IL-18 application significantly reduced IL-4 levels (**Figure 6E**) in the skin and downregulated the transcription of TRPA1 and IL-4RA in the DRG (**Figure 6C**). These findings indicate that reduced IL-18 contributes to aggravated DNFB-induced chronic itch via modulating IL-4/TSLP (indicative of type 2 cytokines) and TRPA1 in *Nlrp3* ^-/-^ mice.

## 4 Discussion

This study reveals that *Nlrp3* knockout aggravates DNFB-induced chronic itch by enhancing type 2 immune responses and TRPA1 transmission. Activation of the NLRP3 inflammasome leads to the production of IL-18, which may mitigate DNFB-induced chronic itch by inhibiting the IL-4/TSLP-TRPA1 axis.

The NLRP3 inflammasome is a crucial component of the innate immune system, triggering the release of IL-1β and IL-18 in response to various stimuli (23, 24). Its activation has been linked to the development of several allergic skin diseases, including vitiligo, urticarial, and psoriasis, and infectious dermatitis such as dermatophytosis and acne (7, 25, 26). Moreover, numerous studies have implicated NLRP3 inflammasome activation in neuropathic and inflammatory pain (27, 28). In the spinal cord, the NLRP3/Casp1/IL-1β inflammasome activation axis is associated with chronic itch in type 2 diabetes and IMQ-induced chronic itch models (12, 29).

It has been speculated that allergen-triggered inflammation and the release of DAMPs are relevant to ACD outcomes and may contribute to NLRP3 inflammasome activation (30). However, the role of NLRP3 and the NLRP3 inflammasome in DNFB-induced chronic itch in the skin, and the underlying mechanisms, remains unclear. Our data demonstrated evidence of signal 1 for NLRP3 inflammasome priming, as indicated by elevated transcription levels of *Nlrp3, Il1b, Il18*, and *Casp1* in the skin of mice with DNFB-induced chronic itch. Additionally, the ASC oligomer, also known as ASC specks, which are essential for assembling the NLRP3 inflammasome and maturing IL-18 and IL-1β, were significantly increased following DNFB challenge, indicating the presence of signal 2 for NLRP3 inflammasome activation in the skin. Deletion of *nlrp3* led to a reduction in IL-18 release but had no effect on IL-1β levels. We speculate that the elevated IL-1β levels, which co-localized with Ly6G, may be produced via an NLRP3-independent mechanism, as suggested by previous studies (18, 19).

Type 2 cytokines, including IL-4, IL-13, and IL-31; keratinocyte-derived TSLP and IL-33; and type 2 immune cells, including Eos, BS, MC, and Th2 cells, along with elevated serum IgE levels, play critical roles in the pathogenesis of AD and ACD (2, 4, 21, 31, 32). IL-4 and IL-13 can act directly on sensory neurons, increasing their sensitivity to various pruritogens and contributing to the persistence of chronic itch in AD (5). Specifically, IL-4 plays a pivotal role in Th2 cell differentiation and is critical for the differentiation and activation of mast cells and basophils during allergen challenges (33).

Additionally, IL-4 induces B-cell activation and class switching to IgE. Th2 cells are the major producers of IL-4. Activated Th cells, predominantly Th2 but also to a lesser extent Th1, Th17, and Th22, release interleukins and other inflammatory mediators that are key markers in AD and chronic itch (34). In our study, the elevated frequencies of immune cells, such as MCs, BSs, Eos, and Th2 cells, may result from the activation or mutual interaction of IL-4 and IL-13 in the skin of DNFB-mediated chronic itch mice. However, in *Nlrp3* deletion mice, higher levels of IL-4 and TSLP expression were observed, which related to enhanced ILC2 and Th2-driven type 2 immune responses. These findings suggest that NLRP3 deletion aggravates DNFB-induced chronic itch via ILC2 and Th2-mediated upregulation of IL-4 and TSLP expression.

TSLP-TSLPR directly activates TRPA1-positive neurons, driving itch behaviors (22). In our study, we observed that TRPA1 expression in the DRG was upregulated in DNFB-treated *Nlrp3^-/-^* mice, whereas TRPV1 expression remained unchanged. Additionally, IL-4 receptors were notably elevated in TRPA1-positive neurons. Administration of the TRPA1 antagonist HC030031 significantly alleviated scratching behavior in DNFB-treated *Nlrp3^-/-^* mice. These findings suggested that increased levels of TSLP and IL-4 act on their receptors in the DRG, triggering TRPA1 activation and aggravating itching. However, further electrophysiological studies or additional antagonist experiments are needed to confirm this conclusion.

The relationship between NLRP3 and type 2 immunity remains controversial. Studies have shown that NLRP3 ligands promote Th1 differentiation through type 2 conventional dendritic cells (35). In patients with AD, low levels of NLRP3 expression in the skin have been correlated with high levels of IL-4 and IL-13 (10). IL-18, a cytokine known to promote Th1 differentiation, plays a key role in this context (36). Our research found that NLRP3 genetic knockout led to reduced IL-18 levels but enhanced Th2 and ILC2-mediated type 2 immunity in a DNFB-induced chronic itch mouse model. Administering recombinant mouse IL-18 reversed severe scratching behavior and reduced IL-4 and TSLP expression during the fourth DNFB challenge in *Nlrp3* deletion mice. These findings suggest that IL-18 protects against DNFB-induced chronic itch in an NLRP3 inflammasome-dependent manner^49^. However, recombinant mouse IL-18 administration significantly reduced scratching behavior only during the fourth DNFB challenge in *Nlrp3* knockout mice. This indicates that a higher dose of rmIL-18 might be needed, or that its effect on reducing scratching behavior may become more evident in the later stages of DNFB-induced chronic itch.

In the nucleus of Th2 cells, NLRP3 binds to the IL-4 promoter and acts as a key transcriptional factor during Th2 differentiation (37). Similarly, in the nucleus of epithelial cells in mice with AD, NLRP3 acts as a critical transcription factor for IL-33 (38). Furthermore, it has been shown that DNCB induces the nuclear localization of NLRP3 in cultured keratinocyte cell lines *in vitro* (39). Therefore, in the skin of DNFB-induced chronic itch mouse models, aside from its role in lowering IL-18, the mechanisms by which NLRP3 or the NLRP3 inflammasome regulates type 2 immunity warrant further investigation, particularly in the context of specific cell types and molecular pathways.

In conclusion, our study demonstrated that *Nlrp3* deletion exacerbates DNFB-induced chronic itch. The underlying mechanisms primarily involve Th2 and ILC2-mediated type 2 immune responses, TSLP and IL-4 expression in the skin, and TRPA1-mediated transmission in the DRG. These findings highlight a protective role of NLRP3-dependent IL-18 activation in mitigating DNFB-induced chronic itch.

## Data Availability

All relevant data is contained within the article: The original contributions presented in the study are included in the article/supplementary material, further inquiries can be directed to the corresponding author/s.
