# Supplementary figures and images for: NLRP3 deficiency aggravated DNFB-induced chronic itch by enhancing type 2 immunity IL-4/TSLP-TRPA1 axis in mice
Source: Front Immunol. 2025 Jan 10;15:1450887. doi: 10.3389/fimmu.2024.1450887 (PMC11758165; doi:10.3389/fimmu.2024.1450887)

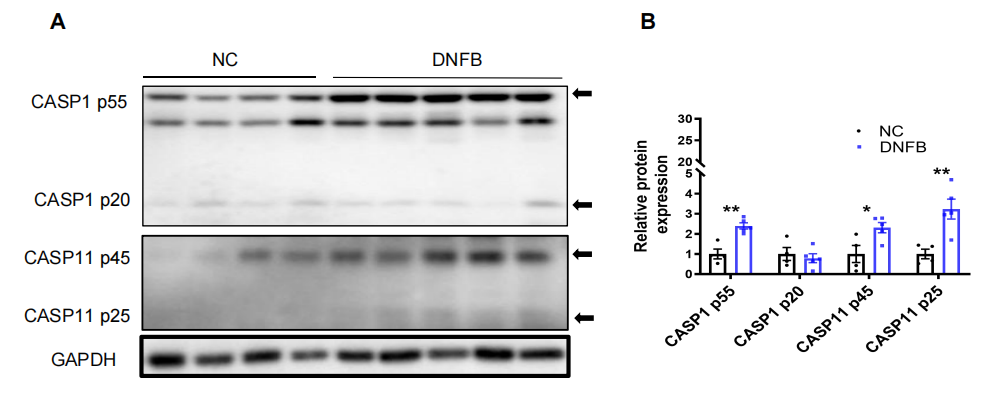

Supplement: Supplementary file 1 [file Image1.tif]

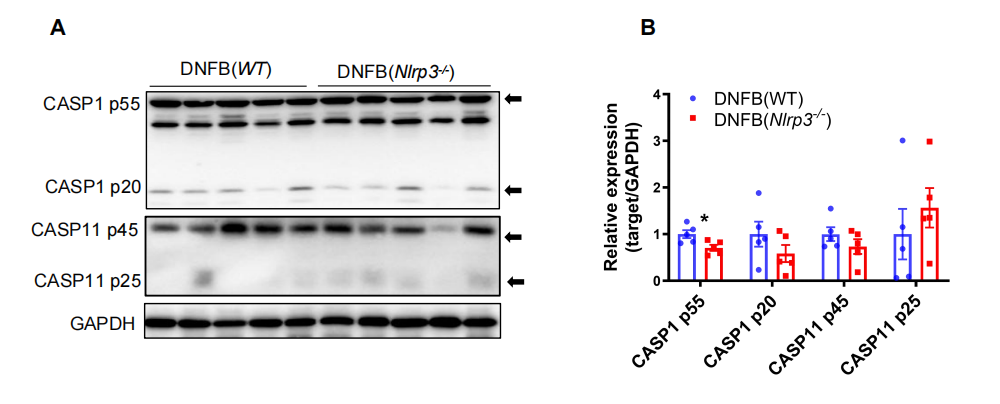

Supplement: Supplementary file 2 [file Image2.tif]

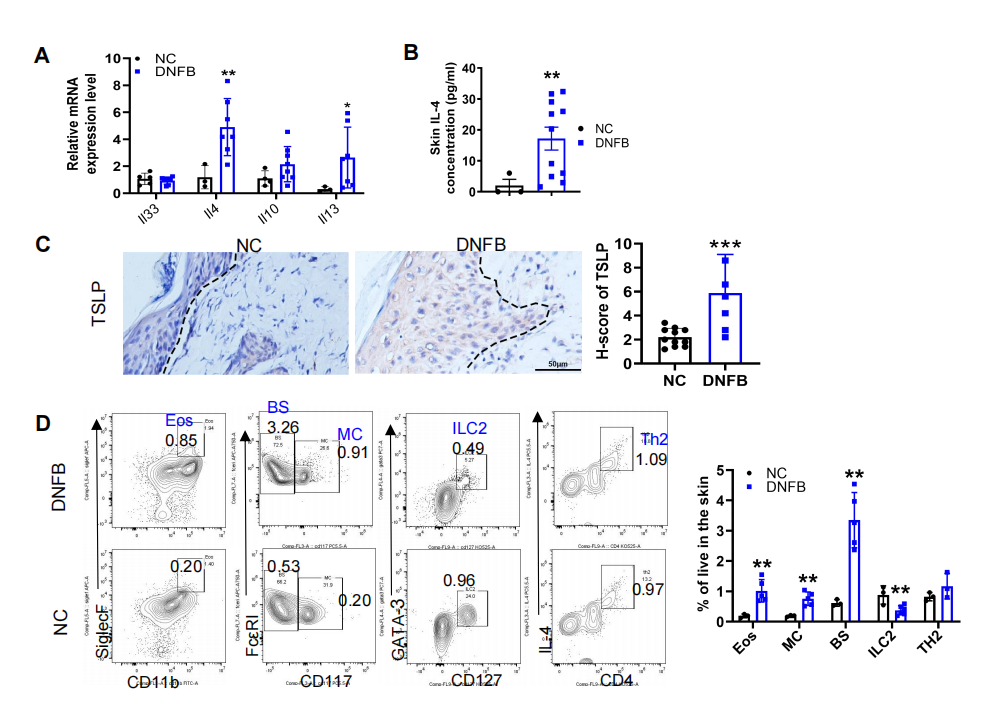

Supplement: Supplementary file 3 [file Image3.tif]

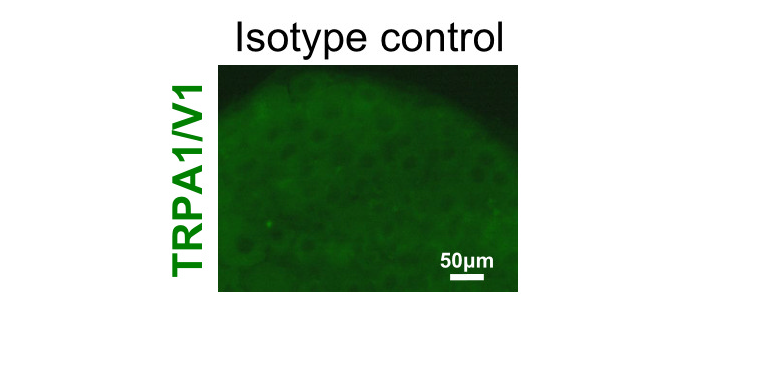

Supplement: Supplementary file 4 [file Image4.tif]

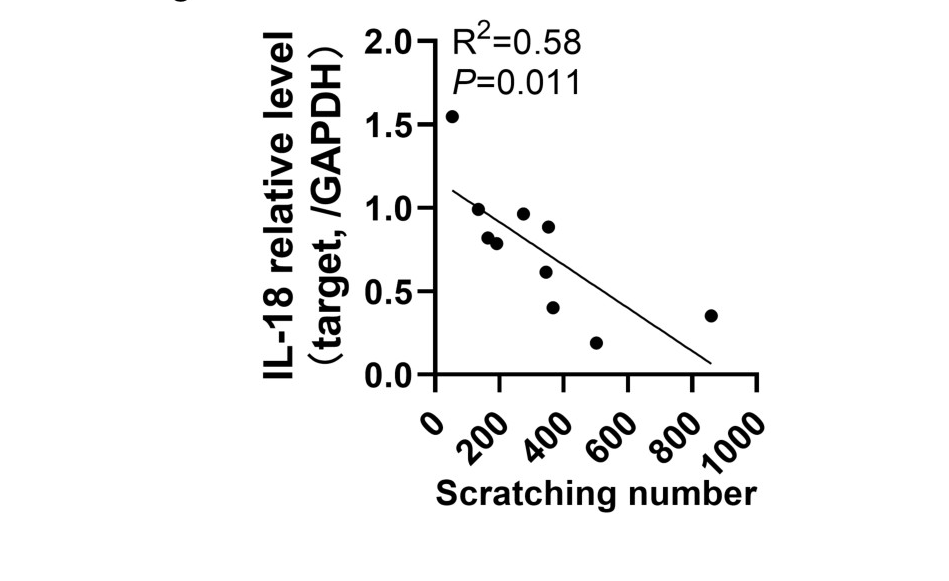

Supplement: Supplementary file 5 [file Image5.tif]
