# Supplementary material for: NLRP3 deficiency aggravated DNFB-induced chronic itch by enhancing type 2 immunity IL-4/TSLP-TRPA1 axis in mice
Source: Front Immunol. 2025 Jan 10;15:1450887. doi: 10.3389/fimmu.2024.1450887 (PMC11758165; doi:10.3389/fimmu.2024.1450887)
Supplement: Supplementary file 6 [file Table1.docx]

**Supplemental Table 1. Primer pairs’ sequences of mice genes used in RT-qPCR**

| **Proteins** | **Genes** | **Forward Primers** | **Reverse Primers** |
| --- | --- | --- | --- |
| GAPDH | *Gapdh* | TGTGTCCGTCGTGGATCTGA | TTGCTGTTGAAGTCGCAGGAG |
| IL-33 | *Il33* | AAGTCAATCAGGCGACGGTG | AGTAGTCCTTGTCGTTGGCA |
| TSLP | *Tslp* | TTCGAGCAAATCGAGGACTGT | TCTTGTTCTCCGGGCAAATGT |
| IL-4 | *Il4* | CGAGCTCACTCTCTGTGGTG | ACCTTGGAAGCCCTACAGACGA |
| IL-10 | *Il10* | CAGTGGAGCAGGTGAAGAGT | AGATGTCAAATTCATTCATGGCCT |
| Il-25 | *Il25* | CTCTCCTTGGAGCTATGAGTTGG | CATGTGGGAGCCTGTCTGTAG |
| IL-13 | *Il13* | AACGGCAGCATGGTATGGAGTG | TGGGTCCTGTAGATGGCATTGC |
| IFN-γ | *Ifng* | GGTCAACAACCCACAGGTCC | CAGCGACTCCTTTTCCGCTT |
| NLRP3 | *Nlrp3* | GATTACCCGCCCGAGAAAGG | GCCTTCTCCTCGCCATTGAA |
| IL-1β | *Il1β* | CCATCCTCTGTGACTCATGGG | TCAGCTCATATGGGTCCGAC |
| AIM2 | *Aim2* | GTCCTCAAGCTAAGCCTCAGA | CACCGTGACAACAAGTGGAT |
| NLRP1a | *Nlrp1a* | GGACCTCATGGTGGTTACTTTC | TCCCAGGGGCCGTAAACTT |
| NLRP1b | *Nlrp1b* | CCTGTGTGACGATGGTGTAAG | AGCCGCAGGATTCTGAGGT |
| NLRC4 | *Nlrc4* | TTGAAGGCGAGTCTGGCAAAG | GGCGCTTCTCAGGTGGATG |
| CASP1 | *Casp1* | CGTCTTGCCCTCATTATCTG | TCACCTCTTTCACCATCTCC |
| CASP11 | *Casp11* | CCTTCTACTCTACAACCCCAC | AAGAGATGACAAGAGCAAGCA |
| IL-18 | *Il18* | TCAAAGTGCCAGTGAACCCC | GGTCACAGCCAGTCCTCTTAC |
| 5-HT | *Htr1a* | CAACAACACCACAACGTCCC | GCTGAAGGTCACGTTGGAGA |
| H1R | *Hrh1* | CAAGACCCGTGCTTCAGCTA | TGAAGTGATGCCAGCCAAGT |
| H4R | *Hrh4* | TCAGGTCCCCTTGGCATTTT | TTCTGTCCACCACAAAGGCT |
| IL-4RA | *Il4ra* | CTAGACACGGAGCTGTCACC | GCTCCAGACCAAGGCATTCT |
| IL-13R | *Il13ra1* | AGAAGCCTAGCCCTTTGGTG | GGGAGCCAGGAACACTTCAT |
| IL-1R | *Il1r1* | ACACGGAGTCATTTGCTGGT | GTTGGGCTGGCATCTGGTAT |
| MrgprA3 | *Mrgpra3* | CCTACCCAAAGGAATTTTTGCCT | GTTGATGTGTGTTCTGGGCG |
| TSLPR | *Crfl2* | AGGCTTCAAGGACTTTACAACAA | TCATGCGAATGAGACTCCCAT |
| TRPA1 | *Trpa1* | TTGGATATTGCAAAGAAGTGATCC | GAGGAACAAGGGCAACACGA |
| TRPV1 | *Trpv1* | TCACCGTCAGCTCTGTTGTC | GATCATAGAGCCTTGGGGGC |

**Abbreviation:** GAPDH, glyceraldehyde-3-phosphate dehydrogenase; NLRP, NOD-, LRR- and pyrin domain-containing protein; IL, interleukin; AIM2, absent in melanoma 2; NLRC4, NLR family CARD domain containing 4; CASP, Caspase; IFN-γ, interferon gamma; TSLP, thymic stromal lymphopoietin; 5-HT, 5-hydroxytryptamine (serotonin) receptor 1A; H1R, histamine receptor H1; IL-4RA, IL-4 receptor alpha;IL-13R, IL-13 receptor alpha 1; IL-1R, IL-1 receptor type 1; MRGPRA3, Mas-related G-protein coupled receptor member A3; *Crfl2*,cytokine receptor like factor 2; TRPA1, transient receptor potential ankyrin 1; TRPV1, transient receptor potential vanilloid 1.
